# Synergistic effect of phenylpropanoids and flavonoids with antibiotics against Gram-positive and Gram-negative bacterial strains

**DOI:** 10.1080/13880209.2024.2389105

**Published:** 2024-08-09

**Authors:** Annamária Kincses, Tasneem Sultan Abu Ghazal, Judit Hohmann

**Affiliations:** a Institute of Pharmacognosy, University of Szeged, Szeged, Hungary; b Interdisciplinary Center for Natural Products, University of Szeged, Szeged, Hungary; c HUN-REN - USZ Biologically Active Natural Products Research Group, University of Szeged, Szeged, Hungary

**Keywords:** Flavones, cinnamic acid derivatives, combinations with antimicrobial drugs, bacterial resistance, adjuvant

## Abstract

**Context:**

The increase in bacterial resistance to currently available medications, which increases mortality rates, treatment costs is a global problem, and highlights the need for novel classes of antibacterial agents or new molecules that interact synergistically with antimicrobials.

**Objective:**

The current work explores the potential synergistic effects of certain natural phenylpropanoids and flavonoids on ciprofloxacin (CIP), ampicillin (AMP), gentamicin (GEN), and tetracycline (TET).

**Materials and methods:**

The adjuvant role of cinnamic acid, *p*-coumaric acid, caffeic acid, ferulic acid, ferulic acid methyl ester, sinapic acid, apigenin, and luteolin was evaluated by determining the MIC (minimal inhibitory concentration) values of antibiotics in the presence of subinhibitory concentrations (200, 100, and/or 50 µM) of the compounds in Gram-positive and Gram-negative bacterial strains using a 2-fold broth microdilution method. The 96-well plates were incubated at 37 °C for 18 h, and dimethyl sulfoxide was used as a solvent control.

**Results:**

The combination of luteolin with CIP, reduced the MIC values of the antibiotic from 0.625 to 0.3125 µM and to 0.078 µM in 100 and 200 µM concentration, respectively, in sensitive *Staphylococcus aureus*. Sinapic acid decreased the MIC value of CIP from 0.625 to 0.3125 µM in *S. aureus*, from 1.56 to 0.78 µM in *Klebsiella pneumoniae*, and the MIC of GEN from 0.39 to 0.095 µM in *Pseudomonas aeruginosa* strains.

**Discussion and conclusions:**

These findings are useful in delaying the development of resistance, as the required antibacterial effect can be achieved with the use of lower concentrations of antibiotics.

## Introduction

The World Health Organization (WHO) considers infectious diseases produced by bacteria, viruses, and fungi to be a global health concern, especially in poor and underdeveloped countries. The emergence of new infectious diseases or the reemergence of old pathogens with new resistance determinants annually accounts for more than 13 million deaths worldwide (Abreu et al. 2017). A major global concern is the increase in bacterial resistance to currently available medications, which emphasizes the need for novel classes of antibacterial agents. Antibiotic adjuvants are substances that increase the efficacy of the present medication (Dhanda et al. 2023). Adjuvants can act with antibiotics on bacterial targets, inhibiting antibiotic resistance directly by circumventing intrinsic resistance mechanisms or improving antibiotic activity in the host (Wright 2016).

One way in which plant-derived compounds exert their antibiotic potential is a positive interaction with antimicrobials. Studies indicate that the use of plant-derived compounds in combination with antibiotics may promote a significant reduction in the minimum inhibitory concentration (MIC) of antibiotics for bacterial strains (Silva et al. 2019). The resulting efficacy is greater than the sum of individual agents, which usually results in an increased or faster killing effect, limiting the potential for the emergence of resistant bacteria. The molecular basis of antibiotic synergy highlights the importance of understanding the mechanisms and primary and secondary targets of antibiotic action; unfortunately, only a few of these data have been reported to date (Wright 2016).

Phenylpropanoids and flavonoids are natural compounds found in many plant families. These compounds can inhibit the growth and activity of a wide range of microorganisms, including clinically significant bacteria, fungi, and food-related strains (Chen 2016; Ruwizhi and Aderibigbe 2020; Liga et al. 2023). They can act as antioxidants due to multiple hydroxyl groups and unsaturated double bonds that react with radicals and oxidative ions in cells. The structure of the benzene or phenol ring in phenolic acids helps them cross cell membranes and exert their biological activities (Hemaiswarya and Doble 2010). Cinnamic acid and its derivatives (*p*-coumaric acid, caffeic acid, ferulic acid, and sinapic acid) based on the structure of C_6_-C_3_ (phenylpropanoid) are widespread phenolic acids in the plant kingdom that can be found in free form in many plants, such as cinnamon, fruits, whole grains, and vegetables (Guzman 2014).

The antimicrobial effects of hydroxycinnamic acids have been evaluated in several studies. Studies have demonstrated the inhibitory effects of caffeic acid, ferulic acid, and sinapic acid on *Listeria monocytogenes*, *Staphylococcus aureus*, *Escherichia coli*, *Bacillus cereus*, and *Salmonella enteritidis* (Borges et al. 2013; Chen 2016; Zhang et al. 2020). The antibacterial effect of caffeic acid is based on inhibiting the bacterial RNA polymerase enzyme. It showed a synergistic effect with fosfomycin on the inhibition of *L. monocytogenes*. It exerted a potentiating effect on antibacterial activity in *S. aureus*, *E. coli*, and *Pseudomonas aeruginosa* when applied in association with norfloxacin, imipenem, and gentamicin, respectively (Lima et al. 2016). Furthermore, it can increase membrane permeability, resulting in the release of cell contents and the access to hydrophobic antibiotics. *p*-Coumaric acid was found to be effective against a variety of pathogen bacteria, including *S. aureus*, *Streptococcus pneumoniae*, *B. subtilis*, *E. coli*, *Shigella dysenteriae*, and *Salmonella typhimurium* (MIC values 10–80 µg/mL). According to Lou et al. (2012), *p*-coumaric acid has two distinct bactericidal mechanisms: it disrupts bacterial cell membranes and binds to bacterial genomic DNA to inhibit cellular functions, ultimately leading to cell death. Sinapic acid (3,5-dimethoxy-4-hydroxycinnamic acid) is an orally bioavailable natural product that was isolated from the methanol extract of yellow mustard seeds. This compound was reported to be effective against *E. coli*, *S. aureus*, and *S. enteritidis* (MIC values 1.8-2.2 mM) (Tesaki et al. 1998).

Apigenin and luteolin are among the most ubiquitous plant flavonoids; their antimicrobial effects have been extensively studied against many bacteria species and their various strains (Farhadi et al. 2019; Wang et al. 2019). Interaction studies revealed substantial results on the synergistic interactions of luteolin and apigenin with levofloxacin in *P. aeruginosa*. According to the study by Hanci and Igan (2023), apigenin showed addictive activity with trimethoprim against *E. coli*.

**Figure 1.**
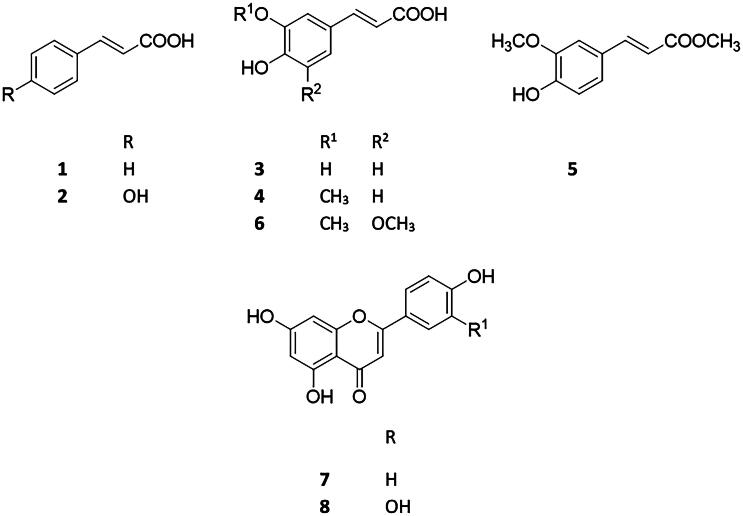
Structures of the investigated compounds **1**–**8**.

The present study aimed to investigate the antibacterial properties of various phenylpropanoids and flavonoids and to analyze their synergistic effects with antibiotics ciprofloxacin (CIP), ampicillin (AMP), gentamicin (GEN), and tetracycline (TET). (*E*)-Cinnamic acid (**1**), (*E*)-*p*-coumaric acid (**2**), (*E*)-caffeic acid (**3**), (*E*)-ferulic acid (**4**), (*E*)-ferulic acid methyl ester (**5**), (*E*)-sinapic acid (**6**), apigenin (**7**) and luteolin (**8**) (Figure 1) were included in the assay to have comparable data for discussion on their effect with antibiotics.

## Materials and methods

### Source of the investigated compounds

(*E*)-Cinnamic acid (**1**) (Sigma-Aldrich, 97%), (*E*)-*p*-coumaric acid (**2**) (Sigma-Aldrich, ≥98%), (*E*)-caffeic acid (**3**) (Sigma-Aldrich, ≥98%), (*E*)-ferulic acid (**4**) (Sigma-Aldrich, ≥99%), (*E*)-sinapic acid (**6**) (Sigma-Aldrich, ≥98%), and luteolin (**8**) (Sigma-Aldrich, ≥98%) were purchased from Merck KGaA (Darmstedt, Germany).

### Isolation of apigenin (7) and ferulic acid methyl ester (5) from Origanum majorana

Apigenin (**7**) and ferulic acid methyl ester (**5**) were isolated from the aerial parts of *Origanum majorana* L. (Lamiaceae). The air-dried plant material (1.5 kg) was extracted by percolation with 17 L MeOH at room temperature MeOH. The extract was concentrated at 1 L and subjected to solvent–solvent partition, with *n*-hexane (1 × 3 L) and CHCl_3_ (1 × 3 L). After evaporation, the CHCl_3_ phase (15.76 g) was separated by open column chromatography (OCC) on a polyamide column (120 g) using H_2_O–MeOH mixtures (6:4, 4:6, 2:8, and 0:1) as eluents, resulting five fractions (Fr. I–V). The fraction V (508 mg) was then subjected to normal phase vacuum liquid chromatography (NP-VLC) using mixtures of *n*-hexane–CHCl_3_ (8:2, 7:3, 1:1, 1:9, 0:100) and CHCl_3_–MeOH (99:1, 98:2, 95:5, 9:1, 7:3, 1:1, 0:100). The combination of fractions collected under thin-layer chromatography (TLC) monitoring guidance yielded eight subfractions (V/1–8). The V/5 (220 mg) was further separated by OCC in polyamide eluted with H_2_O–MeOH (6:4, 1:1, 4:6, 2:8, 100), affording eight subfractions (V/5/a–h). The V/5a was then subjected to gel filtration (GF) on a Sephadex LH-20 with elution of CH_2_Cl_2_–MeOH (1:1), providing nine subfractions (V/5/a/1–9). The fraction V/5/a/7 was subjected to NP-VLC using mixtures of *n*-hexane–CHCl_3_ (2:8, 9:1, 0:100) and CHCl_3_–MeOH (99:1, 95:5, 9:1, 0:100), and then further purified by RP-HPLC on the LiChrospher RP-18 column (250 × 4 mm, 5 μm) using MeCN–H_2_O (1:1, isocratic, 0.8 mL/min) as an eluent, producing apigenin (**7**). Fraction V/6 (138 mg) was subjected to NP-VLC using mixtures of *n*-hexane–CHCl_3_ (2:8, 9:1, 0:100) and CHCl_3_–MeOH (99:1, 95:5, 9:1, 0:100) and further purified by prep NP-TLC on silica gel with cyclohexane–CHCl_3_–acetone (0.5:9:0.5) as the developing system. By this means, (*E*)-ferulic acid methyl ester (**5**) was isolated in pure form. The structures were determined by NMR measurements, and the data were compared with the literature.

*trans*-Ferulic acid methyl ester (**5**): ^1^H NMR (CD_3_OD, 500 MHz) *δ* ppm 3.69 (3H, s, 9-OMe), 3.89 (3H, s, 3-OMe), 6.35 (1H, d, *J* = 15.9 Hz, H-7), 6.80 (1H, d, *J* = 8.2 Hz, H-5), 7.07 (1H, dd, *J* = 8.2, 1.9 Hz, H-6), 7.18 (1H, d, *J* = 1.9 Hz, H-2), 7.61 (1H, d, *J* = 15.9 Hz, H-8) (Masuda et al. 2006).

Apigenin (**7**): ^1^H NMR (CD_3_OD, 500 MHz) *δ* ppm 6.06 (1H, d, *J* = 1.9, H-6), 6.27 (1H, d, *J* = 1.9 Hz, H-8), 6.46 (1H, s, H-3), 6.89 (2H, d, *J* = 8.8 Hz, 3′, 5′), 7.80 (2H, d, *J* = 8.8 Hz, 2′, 6′); ^13^C NMR JMOD (CD_3_OD, 125 MHz) *δ* ppm 96.8 (C-8), 102.4* (C-3), 103.0* (C-6), 103.2 (C-10), 117.4 (C-3′,5′), 122.9 (C-1′), 129.2 (C-2′,6′), 159.9 (C-9), 162.8 (C-5), 163.8 (C-4′), 165.6 (C-7), 183.1 (C-4), * interchangeable signals (Tavakoli et al. 2022).

### Bacterial strains

As Gram-positive strains, *Staphylococcus aureus* ATCC 29213, methicillin- and oxacillin-resistant *S. aureus* MRSA ATCC 43300, *S. epidermidis* ATCC 12228, *Enterococcus faecalis* ATCC 29212, and *Bacillus subtilis* ATCC 6633 were investigated in this study. The Gram-negative strains *Escherichia coli* ATCC 35218, *E. coli* (K-12 AG100, expressing the AcrAB-TolC efflux pump at its basal level), *Salmonella enterica* serovar *typhimurium* SL1344, *Klebsiella pneumoniae* ATCC 700603, and *Pseudomonas aeruginosa* ATCC 27853 were tested. The *Salmonella* strain was kindly provided by Dr. Jessica M. A. Blair (University of Birmingham, Birmingham, UK).

### MIC Determination

The minimum inhibitory concentrations (MICs) of all tested compounds and antibiotics were determined according to the Clinical and Laboratory Standards Institute (CLSI) guidelines (CLSI 2018). 2-fold serial dilutions of compounds at concentrations ranging from 400 µM to 0.78 µM with an adjusted bacterial concentration (5 × 10^5^ CFU/mL) were used to determine the MIC in Mueller Hinton broth (MHB). The turbidity of the bacterial suspension was measured using a McFarland Densitometer (Biosan, Riga, Latvia). The 96-well plates were then incubated at 37 °C for 18 h; at the end of the incubation period, the MIC values of the compounds were determined by visual inspection. DMSO (dimethyl sulfoxide) was used as a solvent control. The values are given as the mean determined for three replicates from three independent experiments. DMSO was tested to ensure there was no antibacterial effect at the concentration (2 v/v%) applied in the test. Ciprofloxacin hydrochloride, ampicillin, gentamicin sulfate, and tetracycline hydrochloride were purchased from Merck KGaA (Darmstadt, Germany).

### Enhancement of the activity of antibiotics

The chemosensitizing activity of compounds **1**-**8** was determined based on the MIC values of the antibiotics in the presence of subinhibitory fixed concentrations of the compounds in both Gram-positive and Gram-negative strains. The tested concentrations of compounds were 200 and/or 100 µM. In cases where a compound showed synergism with an antibiotic at 100 µM, the 50 µM concentration was also tested. MICs were determined in strains using the 2-fold broth microdilution method in 96-well plates, employing serial dilutions of antibiotics (CIP, AMP, GEN, TET). The first four rows contained 2-fold dilutions of antibiotics, and combinations of antibiotics and tested compounds were transferred into the last four rows. The 10^−4^ dilution of the overnight bacterial culture in 50 μL of MHB was then added to each well, except for the medium control wells. Plates were incubated at 37 °C for 18 h. The MIC values of antibiotics and their combinations with tested compounds were determined by visual inspection. The values are given as the mean determined for three replicates from three independent experiments.

## Results

A series of cinnamic acid derivatives with oxygenation scaffolds from un-oxygenated to tri-oxygenated (**1–5**) and flavones (**7**, **8**) that incorporate cinnamic acid part in their structures were investigated to explore their synergistic effects on different types of antibiotics. First, compounds **1**–**8** were tested against five Gram-positive and five Gram-negative bacterial strains. The MIC values of the compounds were more than 400 µM, except for luteolin (**8**), which was active against the *S. epidermidis* ATCC 12228 strain (MIC: 200 µM). The MIC value of luteolin was 400 µM on *S. aureus* ATCC 29213, and *S. aureus* MRSA ATCC 43300 strains (Table 1). Furthermore, the antibiotics selected for the study—CIP, AMP, GEN, and TET, representing the quinolone-, β-lactam-, aminoglycoside-, and tetracycline-type of antibiotics, respectively—were also tested. Most of the bacterial strains used in the study were sensitive to antibiotics, with some exceptions noted in Table 1.

**Table 1.** MIC Values of the tested compounds **1-8** and antibiotics.

Bacteria	MIC (µM)					
	1-7	8	CIP	AMP	GEN	TET
*S. aureus* ATCC 29213	>400	400	0.625	0.78	0.125	0.5
*S. aureus* MRSA ATCC 43300	>400	400	1.25	25	25	0.39
*S. epidermidis* ATCC 12228	>400	200	0.195	3.125	0.03125	100
*E. faecalis* ATCC 29212	>400	>400	0.78	1.56	3.125	25
*B. subtilis* ATCC 6633	>400	>400	0.0625	0.03125	0.024	0.19
*E. coli* ATCC 35218	>400	>400	0.095	5	0.5	1.56
*E. coli* AG100	>400	>400	0.195	6.25	0.39	1.56
*S. Typhimurium* SL1344	>400	>400	0.195	1.56	0.78	1.56
*K. pneumoniae* ATCC 700603	>400	>400	1.56	>500	6.25	12.5
*P. aeruginosa* ATCC 27853	>400	>400	0.78	>500	0.39	12.5

The solvent DMSO had no antibacterial effect (MIC: >2%).

In our study, antibiotic MIC values were determined in the presence of sub-inhibitory concentrations of the compounds (200, 100, and 50 µM) in both Gram-positive and Gram-negative bacteria to assess the chemosensitizing activity of the compounds. The combined effects of antibacterial drugs and phytochemicals are summarized in Tables 2–5.

**Table 2.** Ability of compounds **1-8** to potentiate the activity of ciprofloxacin in bacterial strains^a^.

		MIC reduction									
Cmpd	µM	*S. aureus* ATCC 29213	*S. aureus* MRSA ATCC 43300	*S. epidermidis* ATCC 12228	*E. faecalis* ATCC 29212	*B. subtilis* ATCC 6633	*E. coli* ATCC 35218	*E. coli* AG100	*S.* Typhimurium SL1344	*K. pneumoniae* ATCC 700603	*P. aeruginosa* ATCC 27853
**CIP MIC**		0.625	1.25	0.195	0.78	0.0625	0.095	0.195	0.195	1.56	0.78
**1**	100	None	None	None	None	None	None	None	None	None	None
	200	None	None	None	None	**2-fold**	None	None	None	**2-fold**	None
**2**	100	None	None	None	None	None	None	None	None	None	None
	200	**2-fold**	None	None	None	None	None	None	None	None	None
**5**	100	None	None	None	None	None	None	None	None	None	None
	200	**2-fold**	None	None	None	None	None	None	None	None	None
**6**	100	None	None	None	None	None	None	None	None	None	None
	200	**2-fold**	**4-fold**	None	None	None	None	None	**4-fold**	**2-fold**	None
**7**	50	**2-fold**	**2-fold**	ND	ND	ND	ND	ND	ND	ND	ND
	100	**2-fold**	**4-fold**	None	None	None	None	None	None	None	None
	200	**2-fold**	**4-fold**	ND	None	None	None	None	None	None	None
**8**	50	None	ND	None	ND	ND	ND	ND	ND	ND	ND
	100	**2-fold**	ND	**2-fold**	None	None	None	None	None	None	None
	200	8-fold	ND	ND	None	None	None	None	**2-fold**	None	None

^a^ Compounds **3** and **4** in 100 and 200 µM did not modified the MIC values of CIP. The bold letters indicate a decrease in the MIC of ciprofloxacin; Cmpd: compound; ND: not determined.

**Table 3.** Ability of compounds **1-8** to enhance ampicillin activity in bacterial strains^a^.

		MIC reduction									
Cmpd	µM	*S. aureus* ATCC 29213	*S. aureus* MRSA ATCC 43300	*S. epidermidis* ATCC 12228	*E. faecalis* ATCC 29212	*B. subtilis* ATCC 6633	*E. coli* ATCC 35218	*E. coli* AG100	*S.* Typhimurium SL1344	*K. pneumoniae* ATCC 700603	*P. aeruginosa* ATCC 27853
**AMP MIC**		0.78	25	3.125	1.56	0.03125	5	6.25	1.56	>500	>500
**1**	100	None	None	None	None	None	None	None	None	None	None
	200	None	None	None	None	None	None	None	None	**2-fold**	None
**7**	100	ND	None	**2-fold**	None	None	None	None	None	ND	None
	200	ND	None	ND	None	None	None	None	None	ND	None
**8**	100	ND	ND	**4-fold**	None	None	None	None	None	ND	None
	200	ND	ND	ND	**2-fold**	None	None	None	None	ND	None

^a^ Compounds **2**, **3**, **4**, **5**, and **6** in 100 and 200 µM did not modified the MIC values of AMP. The bold letters indicate a decrease in the MIC of ampicillin; Cmpd: compound; ND: not determined.

**Table 4.** Ability of compounds **1-8** to potentiate the activity of gentamicin in bacterial strains^a^.

		MIC reduction									
Cmpd	µM	*S. aureus* ATCC 29213	*S. aureus* MRSA ATCC 43300	*S. epidermidis* ATCC 12228	*E. faecalis* ATCC 29212	*B. subtilis* ATCC 6633	*E. coli* ATCC 35218	*E. coli* AG100	*S.* Typhimurium SL1344	*K. pneumoniae* ATCC 700603	*P. aeruginosa* ATCC 27853
**GEN MIC**		0.125	25	0.03125	3.125	0.024	0.5	0.39	0.78	6.25	0.39
**1**	100	None	None	None	None	None	None	None	None	None	None
	200	None	None	None	None	**2-fold**	None	None	None	None	None
**5**	50	ND	None	ND	ND	ND	ND	ND	ND	ND	None
	100	None	**2-fold**	None	None	None	None	None	None	None	**2-fold**
	200	None	**2-fold**	None	None	None	None	None	**2-fold**	None	**2-fold**
**6**	50	ND	ND	ND	ND	ND	ND	ND	ND	ND	None
	100	None	None	None	None	None	None	None	None	None	**2-fold**
	200	None	None	None	None	None	None	None	None	None	**4-fold**
**7**	100	ND	ND	None	None	None	None	None	None	ND	None
	200	ND	ND	None	None	None	None	None	None	ND	None
**8**	50	ND	ND	None	ND	ND	ND	ND	ND	ND	None
	100	ND	ND	**2-fold**	None	None	None	None	None	ND	None
	200	ND	ND	ND	None	None	None	None	None	ND	None

^a^ Compounds **2**, **3**, and **4** in 100 and 200 µM did not modified the MIC values of GEN. The bold letters indicate a decrease in the MIC of gentamicin; Cmpd: compound; ND: not determined.

**Table 5.** Ability of compound **1-8** to enhance the activity of tetracycline in bacterial strains^a^.

		MIC reduction									
Cmpd	µM	*S. aureus* ATCC 29213	*S. aureus* MRSA ATCC 43300	*S. epidermidis* ATCC 12228	*E. faecalis* ATCC 29212	*B. subtilis* ATCC 6633	*E. coli* ATCC 35218	*E. coli* AG100	*S*. Typhimurium SL1344	*K. pneumoniae* ATCC 700603	*P. aeruginosa* ATCC 27853
**TET MIC**		0.5	0.39	100	25	0.19	1.56	1.56	1.56	12.5	12.5
**8**	50	None	ND	ND	ND	ND	ND	ND	ND	ND	ND
	100	**2-fold**	None	None	None	None	None	None	None	None	None
	200	2-fold	**2-fold**	ND	None	None	None	None	None	None	None

^a^ Compounds **1**–**7** in 100 and 200 µM did not modified the MIC values of TET. The bold letters indicate a decrease in the MIC tetracycline; Cmpd: compound; ND: not determined.

Natural compounds significantly influenced the potency of CIP to the highest degree. Among the tested combinations, *S. aureus* ATCC 29213 exhibited the highest susceptibility to phenylpropanoids and flavones combined with CIP. Specifically, *p*-coumaric acid (**2**), ferulic acid methyl ester (**5**), and sinapic acid (**6**) demonstrated a 2-fold decrease in the MIC value of CIP when used at a concentration of 200 µM, indicating an improvement in its potency. Apigenin (**7**) and luteolin (**8**) produced the same effect at concentrations of 50 and 100 µM, respectively. It should be noted especially the effect of luteolin (**8**), which reduced the MIC value of CIP from 0.625 to 0.078 µM (8-fold) in *S. aureus* ATCC 29213. Antibacterial tests on *S. aureus* MRSA revealed that combinations of CIP with sinapic acid (**6**) and CIP with apigenin (**7**) were more effective than CIP alone, with the MIC value decreasing from 1.25 to 0.3125 µM (4-fold). Cinnamic acid (**1**) improved the potency of CIP against *B. subtilis*, and luteolin (**8**) increased the effectiveness of CIP on *S. epidermidis*, halving the MIC values in both cases. In relation to Gram-negative bacteria, three combinations exhibited a potentiating effect with CIP against *S. typhimurium* and *K. pneumoniae*. The combination of CIP with sinapic acid (**6**) resulted in a 4-fold reduction, while the combination of CIP with luteolin (**8**) offered a 2-fold reduction in the MIC value of CIP against *S. typhimurium*. The antibacterial potential of CIP against *K. pneumoniae* was increased by both cinnamic acid (**1**) and sinapic acid (**6**), resulting in a 2-fold decrease in the MIC value for CIP (Table 2).

The application of 100 µM of apigenin (**7**) and luteolin (**8**) in combination with AMP reduced the antibiotic MIC value by 2- or 4-fold, respectively, against *S. epidermidis*, demonstrating their potentiating effects. Additionally, luteolin (**8**) at a concentration of 200 µM decreased the MIC of AMP in *E. faecalis* by 2-fold (Table 3).

Combinations of GEN with ferulic acid methyl ester (**5**) showed synergism against the Gram-positive *S. aureus* MRSA at concentrations of 200 and 100 µM. Cinnamic acid (**1**) potentiated the effect of GEN in *B. subtilis.* Regarding Gram-negative bacteria, both ferulic acid methyl ester (**5**) and apigenin (**7**) reduced the MIC value of GEN by 2-fold in *S. thyphimurium*. Furthermore, ferulic acid methyl ester (**5**) was able to modulate the MIC of GEN in *P. aeruginosa*, while sinapic acid (**6**) enhanced the antibacterial effect of GEN, resulting in a 4-fold reduction in the MIC in *P. aeruginosa* (Table 4).

Out of the compounds tested, it was only luteolin (**8**) that demonstrated a notable potentiating effect on TET when tested against strains of *S. aureus*. This effect was evidenced by a significant 2-fold decrease in the MIC of TET (Table 5).

## Discussion

The aim of the present study was to explore the possible synergistic activity of natural phenylpropanoids and flavones with different types of antibiotics commonly used in clinics. Six phenylpropanoids (**1–6**), together with apigenin (**7**) and luteolin (**8**), were tested for their antibiotic potentiating effect. Before evaluating the interactions, an initial antibacterial screening was conducted to determine the MIC values of the tested antibiotics and natural compounds. Luteolin (**8**) was the only compound that exhibited antibacterial activity against both *S. aureus* strains and *S. epidermidis*, with mild MIC values of 200 or 400 µM (Table 1). The previously published antibacterial effects of *p*-coumaric acid (**2**), caffeic acid (**3**), ferulic acid (**4**) and sinapic acid (**6**) were measured in much higher concentration range than in our study (Tesaki et al. 1998; Borges et al. 2013; Chen 2016; Zhang et al. 2020).

The potentiating effect of compounds **1–8** on the activity of four antibiotics (CIP, AMP, GEN, and TET) was assessed using a 2-fold broth microdilution method in 96-well plates. Our study revealed that six of the tested compounds demonstrated the ability to enhance antibiotic activity, resulting in 2-, 4- or 8-fold reductions in MIC values against one resistant strain (*S. aureus* MRSA) and seven susceptible strains (*S. aureus*, *S. epidermidis*, *E. faecalis*, *B. subtilis*, *S. thyphimurium*, *K. pneumoniae*, and *P. aeruginosa*). These effects were observed in both Gram-positive and Gram-negative bacteria, as detailed in Tables 2–5.

The results revealed that the tested compounds were unable to reduce the MIC of all antibiotics in *E. coli* strains. Among the tested combinations, *S. aureus* ATCC 29213 exhibited the highest sensitivity to most of them. The highest potency was demonstrated by luteolin (**8**), which, in combination with CIP and AMP, reduced the MIC values of the antibiotics by 4-fold against *S. epidermidis* (when combined with AMP) and by 8-fold against *S. aureus* ATCC 29213 (when combined with CIP). Similarly, apigenin (**7**) also potentiated the activity of CIP against strains of *S. aureus.* The combination of CIP with 50 µM apigenin (**7**) resulted in a 2-fold reduction in activity, similar to when it was used at concentrations of 100 and 200 µM in the *S. aureus* ATCC 29213 strain. Apigenin (**7**) exhibited the ability to reduce the MIC value of AMP in *S. epidermidis* by 2-fold. Additionally, it demonstrated a similar efficacy with GEN against *S. typhimurium*, also resulting in a 2-fold reduction in the MIC value. The data presented align with previous observations indicating that flavones containing two hydroxyl groups in a meta position in the A-ring possess the ability to enhance the activity of antibiotics (Hummelova et al. 2015).

Among the phenylpropanoids, sinapic acid (**6**) was found to be the most potent, since it enhanced the effects of CIP (Table 2) and GEN (Table 4) when combined with them against three Gram-negative strains (*S. typhimurium*, *K. pneumoniae*, and *P. aeruginosa*) and two Gram-positive strains (*S. aureus* strains), resulting in a 2- to 4-fold reduction in MIC values. Ferulic acid methyl ester (**5**) also increased susceptibility to CIP and GEN against the same microorganisms, reducing MIC values by 2-fold in all cases (Tables 2 and 4). However, caffeic acid (**3**) and ferulic acid (**4**) did not show antibiotic-potentiating effects against any bacteria or in combination with any antibiotics. Lima et al. (2016) reported that caffeic acid can reduce the MIC value of GEN from 625 mg/mL to 24.61 mg/mL. However, such synergism could not be proved in our experiment.

Against the drug-resistant *S. aureus* MRSA strain, combinations of CIP with sinapic acid (**6**) and apigenin (**7**), GEN with ferulic acid methyl ester (**5**) and TET with luteolin (**8**) could modulate the effect of antibiotics. Synergism of flavonoids against this strain was previously investigated. Usman Amin et al. (2016) reported that luteolin (**8**) combined with AMP enhanced the effect of the antibiotic, reducing the MIC value from 128 µg/mL to 64 µg/mL against the *S. aureus* MRSA ATCC 43300 strain and from 162.85 ± 68.05 µg/mL to 81.43 ± 34.02 µg/mL against clinical isolates of MRSA. A similar enhanced effect was found for combinations of luteolin (**8**) with cephradine, ceftriaxone, imipenem, and methicillin. Antibacterial activity against MRSA strains was further enhanced when luteolin (**8**) and antibiotics were used in combination with quercetin (Usman Amin et al. 2016). In the study of Akilandeswari and Ruckmani (2016) combined with apigenin (**7**) significantly reduced the MIC of AMP from 800 µg/mL to 107 µg/mL and the MIC of ceftriaxone from 58 µg/mL to 2.6 µg/mL against MRSA. The findings for inner and outer membrane permeability demonstrated that the combination of apigenin (**7**) with AMP and ceftriaxone damaged the cytoplasmic membrane of MRSA, leading to subsequent leaking of internal components. Electron microscopy clearly demonstrated that the combination significantly damaged the cell wall, morphology, and plasma membrane of the strains (Akilandeswari and Ruckmani 2016). Inhibition of the efflux pump on the drug-resistant bacteria MRSA could also plausibly explain for the co-action of the flavones and antibiotics (Lan et al. 2021).

Antimicrobial activity of sinapic acid (**6**) was previously investigated against a series of human pathogens and foodborne bacteria, including *B. subtilis*, *E. coli*, *Pseudomonas syringae*, *S. aureus*, *Listeria innocua*, *L. monocytogenes*, and *P. fluorescens*, confirming its antibacterial effect with MIC values ranging from 1.9 to 8 mM and 0.2 to 0.7 g/L (Nićiforović and Abramovič 2014). In addition, sinapic acid (**6**) has been studied for its ability to increase antibiotic activity against both sensitive and resistant *Campylobacter jejuni*, a leading bacterial strain causing human gastroenteritis. It was concluded that the synergistic antibacterial activity of sinapic acid (**6**) and other phenylpropanoids in *C. jejuni* is associated with changes in membrane permeability and antibiotic accumulation (Oh and Jeon 2015).

Hemaiswarya and Doble investigated phenylpropanoids **1** to **4** against *E. coli*, *P. aeruginosa*, *S. aureus*, and *Enterobacter aerogenes* in combination with CIP, AMP, and other antibiotics such as amikacin, erythromycin, and vancomycin. According to our study, caffeic acid (**3**) did not show chemosensitizing activity, and *p*-coumaric acid (**2**) only enhanced the activity of *E. aerogenes* when combined with CIP and other antibiotics. Interestingly, cinnamic acid (**1**) and ferulic acid (**4**) increased the antibacterial effect of all antibiotics against *E. coli*, a bacterial strain that was not sensitive to the combinations tested in our experiment. This difference in sensitivity may be attributed to the use of different strains; while Hemaiswarya and Doble (2010) used *E. coli* NCIM 2931, we employed *E. coli* ATCC 35218 and *E. coli* K-12 AG100 strains in our study. Similar differences were observed against *S. aureus*; cinnamic acid (**1**) and ferulic acid (**4**) were ineffective in combination with CIP and AMP in our study against *S. aureus* ATCC 29213 (Tables 2 and 3), but they potentiated antibiotics in Hemaiswarya and Doble’s experiment against the *S. aureus* NCIM 5021 strain.

In our study, considering the detected mode of action of the synergistic activity, different mechanisms can be proposed due to the varied modes of action of the antibiotics used: ciprofloxacin inhibits bacterial DNA synthesis, ampicillin irreversibly inhibits the enzyme transpeptidase, gentamicin inhibits protein synthesis in bacterial cells, and tetracycline inhibits protein synthesis by preventing the attachment of aminoacyl-tRNA to the ribosomal acceptor (A) site. The common mechanism that can be assumed is the impact of compounds **1**, **2**, and **5**–**8** in facilitating the penetration of antibiotics through the bacterial membrane, which acts as a barrier. Changes in membrane permeability and the presence of selective transporters for drug uptake or inhibitors of efflux transporters may be responsible for the higher accumulation of antibiotics in the presence of phenylpropanoids and flavones (Natarajan et al. 2008; Navrátilová et al. 2016; Lan et al. 2021; Fahle et al. 2022).

## Conclusions

The present study has revealed remarkable results on the synergistic interactions of antibiotics with phenylpropanoids and flavones. Cinnamic acid (**1**), *p*-coumaric acid (**2**), ferulic acid methyl ester (**5**), sinapic acid (**6**), apigenin (7) and luteolin (**8**) were found to potentiate antibiotic activity, resulting in 2-, 4-, or 8-fold reductions in MIC values against both resistant (*S. aureus* MRSA) and susceptible (*S. aureus*, *S. epidermidis*, *E. faecalis*, *B. subtilis*, *S. thyphimurium*, *K. pneumoniae* and *P. aeruginosa*) bacterial strains.

Combinations of luteolin (**8**), apigenin (**7**), ferulic acid methyl ester (5) and sinapic acid (**6**) exhibited the greatest enhancement of antibiotic potency, with natural compounds having the most significant influence on CIP potency. Based on data from the literature, these synergistic antibacterial activities may be associated with changes in membrane permeability and antibiotic accumulation.

In terms of structure-activity relationships, it can be observed that cinnamic acid (**1**) without oxygenation on the aromatic ring, coumaric acid (**2**) with a *p*-hydroxy group and caffeic acid (**3**) with 3,4-dihydroxy substitution exhibited limited effectiveness as antibiotic potency enhancers. However, when *p*-coumaric acid and caffeic acid were incorporated in the structures of flavones [apigenin (**7**) and luteolin (**8**)] and condensed with a 5,7-dihydroxylated ring A, the activities were significantly enhanced. A similar increase in the synergistic effect was noted when ferulic acid (**4**) and ferulic acid methyl ester (**5**) was compared (CIP/*S. aureus*, GEN/MRSA, GEN/*S. thyphimurium*, GEN/*P. aeruginosa*). Among the phenylpropanoids, sinapic acid (**6**) with 3,4,5-trioxygenated scaffold exhibited the most potent adjuvant effect, indicating that this oxygenation is favorable for increasing the activity of antibiotics. This is the first report on the antibiotic adjuvant effect of cinnamic acid derivatives in combination with CIP, AMP, and GEN against *S. epidermidis*, *K. pneumoniae*, *S. thyphimurium*, *E. faecalis*, and *B. subtilis*.

These findings are promising in terms of delaying the development of resistance, since achieving the required antibacterial effect may be possible with lower concentrations of antibiotics in the presence of adjuvants.
